# Synapse‐specific expression of calcium‐permeable AMPA receptors in neocortical layer 5

**DOI:** 10.1113/JP271394

**Published:** 2015-12-17

**Authors:** Txomin Lalanne, Julia Oyrer, Adamo Mancino, Erica Gregor, Andrew Chung, Louis Huynh, Sasha Burwell, Jérôme Maheux, Mark Farrant, P. Jesper Sjöström

**Affiliations:** ^1^ Centre for Research in Neuroscience, Department of Neurology and NeurosurgeryThe Research Institute of the McGill University Health Centre, Montreal General HospitalMontrealQuebecCanada; ^2^ Integrated Program in NeuroscienceMcGill UniversityMontrealQuebecCanada; ^3^ Department of Neuroscience, Physiology and PharmacologyUniversity College LondonLondonUK

## Abstract

**Key points:**

- In the hippocampus, calcium‐permeable AMPA receptors have been found in a restricted subset of neuronal types that inhibit other neurons, although their localization in the neocortex is less well understood.
- In the present study, we looked for calcium‐permeable AMPA receptors in two distinct populations of neocortical inhibitory neurons: basket cells and Martinotti cells. We found them in the former but not in the latter. Furthermore, in basket cells, these receptors were associated with particularly fast responses.
- Computer modelling predicted (and experiments verified) that fast calcium‐permeable AMPA receptors enable basket cells to respond rapidly, such that they promptly inhibit neighbouring cells and shut down activity.
- The results obtained in the present study help our understanding of pathologies such as stroke and epilepsy that have been associated with disordered regulation of calcium‐permeable AMPA receptors.

**Abstract:**

AMPA‐type glutamate receptors (AMPARs) lacking an edited GluA2 subunit are calcium‐permeable (CP) and contribute to synaptic plasticity in several hippocampal interneuron types, although their precise role in the neocortex is not well described. We explored the presence of CP‐AMPARs at pyramidal cell (PC) inputs to Martinotti cells (MCs) and basket cells (BCs) in layer 5 of the developing mouse visual cortex (postnatal days 12–21). GluA2 immunolabelling was stronger in MCs than in BCs. A differential presence of CP‐AMPARs at PC‐BC and PC‐MC synapses was confirmed electrophysiologically, based on measures of spermine‐dependent rectification and CP‐AMPAR blockade by 1‐naphtyl acetyl spermine using recordings from synaptically connected cell pairs, NPEC‐AMPA uncaging and miniature current recordings. In addition, CP‐AMPAR expression in BCs was correlated with rapidly decaying synaptic currents. Computer modelling predicted that this reduces spike latencies and sharpens suprathreshold responses in BCs, which we verified experimentally using the dynamic clamp technique. Thus, the synapse‐specific expression of CP‐AMPARs may critically influence both plasticity and information processing in neocortical microcircuits.

## Key points

- In the hippocampus, calcium‐permeable AMPA receptors have been found in a restricted subset of neuronal types that inhibit other neurons, although their localization in the neocortex is less well understood.
- In the present study, we looked for calcium‐permeable AMPA receptors in two distinct populations of neocortical inhibitory neurons: basket cells and Martinotti cells. We found them in the former but not in the latter. Furthermore, in basket cells, these receptors were associated with particularly fast responses.
- Computer modelling predicted (and experiments verified) that fast calcium‐permeable AMPA receptors enable basket cells to respond rapidly, such that they promptly inhibit neighbouring cells and shut down activity.
- The results obtained in the present study help our understanding of pathologies such as stroke and epilepsy that have been associated with disordered regulation of calcium‐permeable AMPA receptors.

**Abbreviations**
aCSF: artificial cerebrospinal fluid
AMPAR: α‐amino‐3‐hydroxy‐5‐methyl‐4‐isoxazolepropionic acid receptor
AP: action potential
AP5: 2‐amino‐5‐phosphonovaleric acid
BC: basket cell
CI: calcium‐impermeable
CP: calcium‐permeable
DAPI: 4′,6‐diamidino‐2‐phenylindole
*E*_rev_: reversal potential
IN: inhibitory neuron
*I*–*V*: current–voltage
L: layer
MC: Martinotti cell
mEPSC: miniature EPSC
MGE: medial ganglionic eminence
Naspm: 1‐naphtyl acetyl spermine
NBQX: 2,3‐dihydroxy‐6‐nitro‐7‐sulphamoyl‐benzo[f]quinoxaline‐2,3‐dione
NMDAR: NMDA receptor
NPEC: 1‐(2‐nitrophenyl)ethoxycarbonyl
PBS: phosphate‐buffered saline
PC: pyramidal cell
2PLSM: 2‐photon laser‐scanning microscopy
Pvalb: parvalbumin
RI: rectification index
ROI: region of interest
spm: spermine
Sst: somatostatin
WT: wild‐type

## Introduction

Calcium transients critically regulate synapse development, functioning and plasticity. The involvement of NMDA receptors (NMDARs) and voltage‐gated calcium channels in mediating these transients is well established (Sjöström & Nelson, 2002; Sjöström *et al*. 2008; Maheux *et al*. 2015). In addition, α‐amino‐3‐hydroxy‐5‐methyl‐4‐isoxazolepropionic acid receptors (AMPARs) either containing an unedited version of the GluA2 subunit or lacking it completely are also calcium‐permeable (CP) (Hume *et al*. 1991) and are able to trigger long‐term plasticity (Kullmann & Lamsa, 2007).

NMDARs are blocked by extracellular Mg^2+^ ions at resting and hyperpolarized membrane potentials, whereas GluA2‐lacking CP‐AMPARs are blocked by endogenous intracellular polyamines at depolarized potentials (Bowie & Mayer, 1995; Donevan & Rogawski, 1995; Kamboj *et al*. 1995; Koh *et al*. 1995). This results in a characteristic inwardly rectifying current–voltage (*I*–*V*) relationship, which can be used to identify CP‐AMPARs. Subunit composition also determines the kinetic properties of AMPARs. For example, CP‐AMPARs typically have faster desensitization rates than GluA2‐containing AMPARs (Hume *et al*. 1991; Traynelis *et al*. 2010; Sobolevsky, 2015), as well as a higher single‐channel conductance (Swanson *et al*. 1997).

In the hippocampus, several studies have identified CP‐AMPARs at excitatory inputs onto PCs in early development, under certain pathological conditions, and transiently after long‐term potentiation (Plant *et al*. 2006; Rozov *et al*. 2012; Mattison *et al*. 2014; but see also Lu *et al*. 2009). Under physiological conditions, however, CP‐AMPARs are primarily associated with excitatory inputs onto inhibitory neurons (IN), where they contribute to the induction of synaptic long‐term plasticity (Lamsa *et al*. 2007 *b*; Camire & Topolnik, 2014). However, the precise pattern of expression of CP‐AMPARs in neocortical INs is not known. One reason for this may be the complexity of the neocortical circuitry because neocortical IN classification remains a challenge (DeFelipe *et al*. 2013). INs are generally classified by morphology, firing pattern and genetic markers (Markram *et al*. 2004; Ascoli *et al*. 2008; DeFelipe *et al*. 2013; Kepecs & Fishell, 2014), such as parvalbumin (Pvalb) and somatostatin (Sst) (Toledo‐Rodriguez *et al*. 2005). In layer (L)5, two key IN types are fast‐spiking Pvalb‐positive basket cells (BCs) and Sst‐expressing Martinotti cells (MCs) that have a characteristic accommodating spiking pattern. These IN types have strikingly different morphologies: classically, BC axons are largely intralaminar (but see Buchanan *et al*. 2012), whereas MC axons ascend and ramify extensively up to L1 (Kawaguchi & Kubota, 1996, 1997; Markram *et al*. 2004; Silberberg & Markram, 2007; Buchanan *et al*. 2012). Excitatory inputs onto these two IN types also have very different short‐term dynamics: those onto MCs short‐term facilitate, whereas those onto BCs rapidly depress once activated (Silberberg & Markram, 2007; Buchanan *et al*. 2012; Blackman *et al*. 2013). As a consequence of these distinct features, BCs and MCs are relatively easy to distinguish compared to other neocortical IN types. In addition, neocortical BCs are also the most numerous, accounting for approximately half of all neocortical INs (Markram *et al*. 2004), whereas MCs compensate for their lower numbers by strongly and efficiently inhibiting PCs (Berger *et al*. 2010).

The above‐described differences in morphology and synaptic properties have important implications for MC and BC function in the local circuit. The strong facilitation of excitatory inputs onto MCs, for example, enables delayed‐onset feedback inhibition (Silberberg & Markram, 2007), which increases rapidly with the number of excitatory synapses recruited so that PCs can via MCs efficiently limit their own spiking activity (Kapfer *et al*. 2007; Berger *et al*. 2010). MCs specifically inhibit PC dendrites, and are particularly efficient at shutting down dendritic calcium spikes and spiking output, as well as plasticity in PCs (Murayama *et al*. 2009; Bar‐Ilan *et al*. 2012; Gidon & Segev, 2012). The short‐term depressing excitatory inputs onto BCs, on the other hand, ensure that this cell type mediates early‐onset feed‐forward inhibition of PCs. This occurs predominantly perisomatically (Kawaguchi & Kubota, 1997; Buchanan *et al*. 2012), where BC inhibition effectively shortens the integrative time window for excitation (Pouille & Scanziani, 2001; Mittmann *et al*. 2005). In combination, early‐onset perisomatically targeting BCs and late‐onset dendritically targeting MCs can thus remap a temporal high‐frequency pattern of excitation into a spatial pattern of inhibition, such that the soma is inhibited first, followed by dendrites later (Pouille & Scanziani, 2004; Blackman *et al*. 2013). We have recently demonstrated that presynaptic NMDARs enhance the delayed‐onset MC‐mediated feedback inhibition of PCs by specifically boosting PC inputs to MCs during high‐frequency firing (Buchanan *et al*. 2012). Whether specific glutamate receptor types similarly assist in early‐onset BC‐mediated inhibition is not known.

In the present study, we looked for CP‐AMPARs at synapses from PCs onto BCs and MCs in L5 of the mouse visual cortex. We found that CP‐AMPARs were expressed at PC‐BC but not at PC‐MC synapses. We also observed that CP‐AMPAR‐containing synapses onto BCs were associated with significantly more rapid decay kinetics, which helps to shorten spike latencies and sharpen the suprathreshold response duration in BCs. We propose that synaptic CP‐AMPARs in BCs, by virtue of their relatively rapid kinetics, may narrow the BC‐controlled integration time window for excitation in PCs (Pouille & Scanziani, 2001), temporally sharpening information processing in cortical microcircuits.

## Methods

### Ethics

All procedures conformed to the standards and guidelines set in place by the *UK Animals (Scientific Procedures) Act 1986* and the *Canadian Council on Animal Care*, with appropriate licenses. Mice were anaesthetized with isoflurane and killed once the hind‐limb withdrawal reflex was lost. Transgenic animals had no abnormal phenotype. Every attempt was made to ensure minimum discomfort to the animals at all times.

### Animals

Experiments were performed using tissue from mice aged between postnatal days 12 and 21 (P12–21). Most recordings were obtained from C57BL/6 wild‐type (WT) mice. To target MCs genetically, we employed the GIN mouse line (Jackson Labs 3718; Jackson Laboratories, Bar Harbor, ME, USA) (Oliva *et al*. 2000). To target BCs genetically, we used the G42 mouse line (Jackson Labs 7677) (Chattopadhyaya *et al*. 2004).

### Immunolabelling

P21 WT mice were anaesthetized with isoflurane and transcardially perfused with 0.1 m phosphate‐buffered saline (PBS) (pH 7.4) followed by 4% paraformaldehyde. Brains were removed and postfixed overnight in 4% paraformaldehyde and transferred to 10% sucrose (w/v). Brains were dissected and 40 μm thick frozen sections of visual cortex were cut using a sliding microtome (HM 450; Thermo Scientific, Waltham, MA, USA) and collected in PBS. Slices were incubated for 90 min in a blocking solution containing 20% normal goat serum (v/v), 1% BSA (w/v) and 0.5% Triton (v/v). Slices were then incubated overnight at 4°C with the primary antibodies, mouse anti‐Pvalb 235 at 1:500 (Swant Inc., Marly, Switzerland), rat anti‐Sst MAB354 at 1:100 (Millipore, Billerica, MA, USA) and rabbit anti‐GluA2 GluR2C‐Rb‐Af1050 at 1:200 (Frontier Institute Ltd, Hokkaido, Japan), in a carrier solution containing 2% normal goat serum, 1% BSA and 0.5% Triton. Of note, the anti‐GluA2 antibody is selective because it shows a complete loss of labelling in brains from GluA2 knockout mice (Yamasaki *et al*. 2011). Slices were washed in carrier solution and incubated for 1 h at room temperature with the secondary antibodies (1:250). Secondary antibodies were: Alexa Fluor 488 Goat Anti‐Mouse IgG 115‐545‐062 (Jackson ImmunoResearch, West Grove, PA, USA), Alexa Fluor 647 Goat Anti‐Rabbit IgG 111‐175‐144 (Jackson Immuno Research), Alexa Fluor 555 Goat Anti‐Rat IgG A‐21434 (Life Technologies, Grand Island, NY, USA) and Alexa Fluor 488 Goat Anti‐Rabbit IgG A‐11008 (Life Technologies). Slices were again washed three times, incubated in 4′,6‐diamidino‐2‐phenylindole (DAPI) (1:1000) at room temperature for 10 min and washed in PBS for 15 min before being mounted using anti‐Fade gold (P36930; Invitrogen, Carlsbad, CA, USA) and kept in the dark at 4°C until imaging. Primary and secondary antibodies were initially tested for optimal dilution, with reference to previously published studies (Shimuta *et al*. 2001; Fukaya *et al*. 2006; Xu *et al*. 2006; Gonchar *et al*. 2007; Cammalleri *et al*. 2009; Antonucci *et al*. 2012; Leon‐Espinosa *et al*. 2012; Massi *et al*. 2012; Huang *et al*. 2013). To avoid fluorescence cross‐talk, fluorophores were imaged sequentially using a confocal microscope (SPE; Leica Microsystems, Wetzlar, Germany).

Analysis of antibody labelling was performed manually using Fiji (Schindelin *et al*. 2012). In each individual image stack, L5 boundaries were identified by the presence of labelled PCs in the GluA2 channel. To quantify fluorescence intensity across labelled cells, the mean grey value in selected regions of interest (ROIs; ∼3 μm in diameter) centred on the brightest regions of the cell body was measured. ROIs of the same area were used to determine the average mean grey background (average of 15 values for each stack) and subtracted from all measurements. The GluA2 labelling in PCs was used to normalize IN GluA2 intensity across slices and animals.

### Acute slice preparation

As described previously (Sjöström *et al*. 2001; Buchanan *et al*. 2012), the brain was rapidly removed and placed in <4°C artificial cerebrospinal fluid (aCSF) containing (in mm): 125 NaCl, 2.5 KCl; 1 MgCl_2_; 1.25 NaH_2_PO_4_; 2 CaCl_2_; 26 NaHCO_3_ and 25 d‐glucose and bubbled with 95% O_2_/5% CO_2_, adjusted to 338 mOsm with glucose). Three‐hundred micron thick near‐coronal slices were cut from visual cortex with a Leica VT1200S or a Campden Instruments (Loughborough, UK) 5000 mz‐2 vibratome. Slices were heated to 32°C for ∼15 min and were subsequently left to cool to room temperature for >1 h before being transferred to the recording chamber. To improve slice quality, in some cases, dissection was carried out with partial replacement of Na^+^ and with elevated Mg^2+^ concentration, using a solution containing (in mm): 87 NaCl, 75 sucrose, 2.5 KCl, 7 MgSO_4_, 1.25 NaH_2_PO_4_, 0.5 CaCl_2_, 26 NaHCO_3_ and 25 d‐glucose.

### Electrophysiology

#### General electrophysiological methods

Neurons were patched with infrared video Dodt contrast using 40× objectives and customized microscopes (SliceScope; Scientifica Ltd, Uckfield, UK). The medial side of primary visual cortex was targeted based on the presence of a granular L4. To target MCs genetically, we used slices from the GIN mouse line (Jackson Labs 3718) (Oliva *et al*. 2000), whereas BCs were targeted genetically using the G42 mouse line (Jackson Labs 7677) (Chattopadhyaya *et al*. 2004). BCs and MCs were most often targeted by the rounded non‐pyramidal appearance of somata in slices from C57BL/6 WT mice. L5 PCs were targeted by their large pyramidal somata and characteristic thick apical dendrite. IN cell identity was always verified *post hoc* by manual reconstruction and morphometry (Fig. 2). All recordings were made in L5, as determined by the presence of the conspicuously large somata of L5 PCs.

Whole‐cell recordings were obtained using BVC‐700A (Dagan Corporation, Minneapolis, MN, USA) or MultiClamp 700B amplifiers (Molecular Devices, Sunnyvale, CA, USA). Voltage and current signals were filtered at 4–10 kHz and acquired at 10–20 kHz using PCI‐6229 boards (National Instruments, Austin, TX, USA) and custom software (Sjöström *et al*. 2001) running in Igor Pro, version 6.37 (WaveMetrics Inc., Lake Oswego, OR, USA). Patch pipettes were pulled from medium‐wall capillaries using a P‐97 or P‐1000 electrode puller (Sutter Instruments, Novato, CA, USA).

#### Paired recordings

Presynaptic PCs were patched with pipettes (4–6 MΩ) filled with a gluconate‐based current‐clamp solution containing (in mm): 5 KCl, 115 K‐gluconate, 10 K‐Hepes, 4 Mg‐ATP, 0.3 Na‐GTP, 10 Na_2_‐phosphocreatine and 0.02–0.04 Alexa Fluor 594, adjusted to pH 7.2–7.4 with KOH and to 310 mOsm with sucrose. Postsynaptic cells were patched with a caesium‐based voltage‐clamp solution containing (in mm): 100 Cs‐gluconate, 5 CsCl, 10 Hepes, 4 Mg‐ATP, 0.3 Na‐GTP, 10 Na_2_‐phosphocreatine, 8 NaCl, 5 QX‐314‐Cl, 5 TEA‐Cl, 0.02 Alexa Fluor 594 and 0.1 spermine tetrahydrochloride, adjusted to pH 7.2–7.4 with CsOH and to 310 mOsm with sucrose. In some recordings, the internal solution included 0.1% w/v biocytin. When specified, 200 μm 1‐naphtyl acetyl spermine (Naspm) (Santa Cruz Biotechnology, Santa Cruz, CA, USA) or 200 μm 2‐amino‐5‐phosphonovaleric acid (AP5) (Sigma, St Louis, MO, USA) was bath applied. Because neocortical connectivity is sparse (Song *et al*. 2005), we used quadruple recordings to rapidly find synaptically connected neuronal pairs (Sjöström *et al*. 2001, 2003). To assess connectivity, five action potentials (APs) were elicited at 30 Hz in the presynaptic cell every 10–15 s by 5 ms long ∼1.3 nA current injections, and 10–20 traces were averaged. In rectification experiments, postsynaptic cells were clamped for 6–10 s at APs ranging from –100 to +50 mV at the same time as evoking two to five APs at 30 Hz in the presynaptic PC. Each voltage step was repeated six to 20 times every 10–15 s. With Naspm wash‐in, the postsynaptic cell was held at –80 mV to minimize blockade by intracellular spermine. Series resistance was monitored but not compensated, as described by Mahanty & Sah (1998). We verified that series resistance was not different across postsynaptic cell type (PCs: 19 ± 2 MΩ, *n* = 3; BCs: 23 ± 1 MΩ, *n* = 14; MCs: 24 ± 2 MΩ, *n* = 10; ANOVA *P* = 0.16). In experiments measuring synaptic current decay time constants (Fig. 9 *A*), we verified that we did not have spurious differences in animal age or perfusion temperature that could potentially explain the differences in decay kinetics (age in postnatal days, PCs: 14 ± 0.8; BCs: 13 ± 0.2; MCs: 14 ± 0.5; ANOVA *P* = 0.22; perfusion temperature, PCs: 32 ± 0.1°C; BCs: 32 ± 0.05°C; MCs: 32 ± 0.1°C; ANOVA *P* = 0.72) (Fig. 9 *A*).

#### Miniature EPSC (mESPC) recordings

We recorded mEPSCs from BCs in the presence of 20 μm AP5, 20 μm SR‐95531, 1 μm CGP 54626 and 0.5 μm TTX‐citrate. To block potassium channels and improve the voltage clamp, aCSF was supplemented with 4 mm TEA‐Cl in some recordings. Patch pipettes (3–6 MΩ) were filled with the gluconate current‐clamp solution (see above) or a voltage‐clamp solution containing (in mm): 100 Cs‐gluconate, 5 CsCl, 10 Hepes, 2 Mg‐ATP, 0.3 Na‐GTP, 10 Na_2_‐phosphocreatine, 8 NaCl, 5 QX‐314‐Cl, 5 TEA‐Cl, 20 K_2_‐ATP, 0.2 EGTA and 0.02 Alexa Fluor 594, adjusted to pH 7.2–7.4 with CsOH and to 310 mOsm with sucrose. The internal solution also included 0.1% w/v biocytin in some cases. High K_2_ATP was present to buffer endogenous spermine. In some recordings, 0.5 mm spermine tetrahydrochloride was added to yield a free internal spermine concentration of ∼40 μm (Rozov *et al*. 2012). For rectification measurements, we used the voltage‐clamp internal solution and mEPSCs were recorded at –60 mV and +60 mV. With Naspm wash‐in, we used current‐clamp internal solution, and BCs were voltage‐clamped at –60 mV throughout. The use of current‐clamp solution enabled us to determine intrinsic cellular properties: 500 ms long current steps ranging from –200 to +700 pA were injected at 40 pA increments. Here, cells were only included if the resting membrane potential was –65 mV or less.

#### Dynamic clamp

Conductance clamp experiments were implemented using a second electrophysiology rig computer as a slave, similar to that described previously (Kemenes *et al*. 2011; Yang *et al*. 2015). To simulate the synaptic current *i*_syn_(*t*) = *g*_syn_(*t*)*[*E*_rev_ – V_m_(*t*)] in real‐time, the slave computer ran a custom script in Igor Pro (WaveMetrics Inc.) that read two analogue‐to‐digital inputs (the *g*_syn_ command from the rig computer and the *V*_m_ voltage reading from the amplifier) and wrote one digital‐to‐analogue output (the *i*_syn_ current command to the amplifier) of a PCI‐6229 board (National Instruments) at maximal non‐synchronized speed using an infinite loop. With NIDAQTools MX, version 1.06 (WaveMetrics Inc.), Igor Pro, version 6.37 (WaveMetrics Inc.), 32‐bit Windows 7 (Microsoft, Redmond, WA, USA) and a Rack Mount Industrial PC model SL‐4U‐CL‐LLQ35‐HA (2.66 GHz Core 2 Quad Processor, 1333 MHz front‐side bus) (SuperLogics, Natick, MA, USA), we reliably obtained steady sampling rates close to 30 kHz, effectively achieving real‐time dynamic clamp. The conductance waveform *g*_syn_(*t*) was determined by the master computer and was defined as a double exponential (compare ‘Computer modelling’ below) with a fast time constant τ_rise_ = 1.4 ms and a slow time constant τ_decay_ of either 3 ms or 5 ms, which was close to the synaptic current kinetics that we found in BCs and MCs (Table 1). In reality, AMPAR synaptic conductances rise and decay faster than this; dendritic cable filtering made our time constant measurements overestimations. However, because we injected the conductances into the soma, these filtered kinetics were more realistic as they accounted for dendritic cable filtering.

**Table 1:** **Synaptic properties of BCs and MCs**

Experiment	Parameter	BC	*n*	MC	*n*	*P*
Paired recording EPSCs	Paired‐pulse ratio	0.36 ± 0.03	14	5.2 ± 1	10	***
	τ_rise_ (ms)	1.8 ± 0.3	22	1.9 ± 0.3	10	NS
	τ_decay_ (ms)	2.9 ± 0.2	22	5.1 ± 0.6	10	**
	τ_decay_ in AP5 (ms)	2.8 ± 0.3	6	5.2 ± 0.6	3	**
	*E*_rev_ (mV)	14 ± 2	14	4.6 ± 3	10	*
AMPA uncaging EPSCs	τ_rise_ (s)	0.16 ± 0.02	33	0.12 ± 0.02	20	NS
	τ_decay_ (s)	1.0 ± 0.1	33	1.7 ± 0.1	20	***
	τ_decay_ in Naspm (s)	1 ± 0.1	7	1.6 ± 0.2	7	**
	*E*_rev_ (mV)	16 ± 2	19	6.4 ± 2	10	*

Data were taken from BCs and MCs in **Figs** 5, 7 and 9. AMPA uncaging EPSC *E*_rev_ was indistinguishable in BCs with and without internal spermine (with: 17 ± 3 mV, *n* = 11; without: 15 ± 4 mV, *n* = 8; *P* = 0.67) and data were pooled here. The same was true for BC uncaging τ_decay_ (for statistics, see **Fig**. 9***B***) and τ_rise_ (with spermine: 0.20 ± 0.03 ms, *n* = 11; without: 0.12 ± 0.07 ms, *n* = 8; *P* = 0.29). NS, not significant.

BCs were patched as described for paired recordings. We systematically altered the peak conductance, *g*_syn_, according to a bisection algorithm to find the first and second rheobase conductance values, *g*_rheo1_ and *g*_rheo2_, defined as the lowest conductance values for which one and two spikes, respectively, were obtained (compare ‘Computer modelling’). This procedure was thus repeated once each for the two time constant values, τ_2_ = 3 ms and τ_2_ = 5 ms, where the former value emulated a BC excitatory input, whereas the latter value simulated a slow‐decaying excitatory input to an MC but in the same recorded BC. Our experimental design removed other contributing factors such as filtering by the membrane time constant, τ_M_, or differences in short‐term plasticity (Buchanan *et al*. 2012) and focused solely on the role of excitatory synaptic input kinetics. Working with rheobase conductance values enabled across‐cell comparisons, as well as comparisons with the computer model (see ‘Computer modelling’ below) (Fig. 10). With τ_2_ = 3 ms as for a CP‐AMPAR‐mediated input to a BC, we obtained *g*_rheo1_ = 1 ± 0.2 nS and *g*_rheo2_ = 3.7 ± 0.9 nS (*n* = 5 cells). With τ_2_ = 5 ms as for a calcium‐impermeable (CI)‐AMPAR‐mediated input to an MC, we obtained *g*_rheo1_ = 0.8 ± 0.1 nS and *g*_rheo2_ = 2 ± 0.5 nS (the same *n* = 5 cells). In Fig. 10 *Bii*, we opted to sidestep the electrophysiologist's sign convention that amplifier current injections are represented as upward positive deflections, instead illustrating these as downward negative deflections to simplify comparison with the computer simulation shown in Fig. 10 *Aii*.

### Analysis of electrophysiological data

Stability criteria were applied to all recordings: membrane potential was not allowed to vary by more than 8 mV, input resistance not by more than 30% and temperature had to remain within 31–33°C throughout the recordings. If not, the recordings were discarded or truncated. Experiments with unstable baseline, as assessed using a *t* test of Pearson's *r* at the *P* < 0.05 significance level, were discarded. Input resistance was measured by a 250 ms long test pulse of –50 pA in current clamp, or –25 mV in voltage clamp.

In paired recordings, the AMPA current was measured at a 1 ms long window positioned at the peak of the first EPSC in a train, whereas the NMDA current was measured 20 ms later. In paired recordings and in uncaging experiments, we defined the rectification index, RI_slope_, as a ratio of *I*–*V* slopes (Adesnik & Nicoll, 2007; Jackson *et al*. 2011). For each recording, we first applied linear regression to *I*–*V* data for which AMPAR current was less than zero, which gave a slope, *s*1, as well as an AMPAR reversal potential, *E*_rev_. Next, we fit *I*–*V* data for which voltages were greater than *E*_rev_ with a line constrained to intersect the x‐axis at *E*_rev_. This gave a second slope, *s*2. The RI_slope_ was then calculated as *s*2/*s*1. The RI_slope_ metric had the benefit that it accounted for *E*_rev_ variations across recordings (Adesnik & Nicoll, 2007), as well as across cell types (Table 1). The robustness of the RI_slope_ was verified in BC AMPA uncaging experiments without internal spermine (Fig. 7). To average *I*–*V* curves across cells, current values were normalized to the value at –60 mV. To quantify the effect of Naspm on PC‐BC connections, we determined the ratio of the amplitude of the first EPSC in a train during Naspm wash‐in over that during the baseline. Liquid junction potential (10 mV) was accounted for in the off‐line analysis.

Analysis of mEPSCs was performed using NeuroMatic, version 2.8 (http://www.neuromatic.thinkrandom.com) running in Igor Pro. For detection, records were digitally low‐pass Butterworth filtered at 2 kHz, and events were detected using threshold crossing of 2.5 SDs over background noise (Kudoh & Taguchi, 2002), which corresponded to 8.0 ± 0.4 pA (*n* = 23). All automatically detected events were individually visually inspected and manually triaged. To limit the influence of dendritic filtering, analysis was restricted to events with 20–80% rise times faster than 0.4 ms. To reduce error in estimating the rise time and charge of noisy mEPSCs, individual events were fitted with an empirical equation and measures were taken from the fit waveform (Bekkers & Stevens, 1996; Bekkers & Clements, 1999). We calculated mEPSC rectification as the ratio of the summed mEPSC charge (i.e. the sum of mEPSC charge from equal lengths of recordings at positive and negative voltages); RI_+60/–60_. The effect of Naspm on summed mEPSC charge was assessed by comparing 100 ms long epochs at the beginning of the recording and 20 min after Naspm wash‐in. RI_+60/–60_ was adjusted for liquid junction potential (11 mV).

### Optical methods

#### Two‐photon imaging

Two‐photon excitation was achieved using a Chameleon XR (Coherent, Santa Clara, CA, USA) or MaiTai BB (Spectraphysics, Santa Clara, CA, USA) Ti:Sa laser, tuned to 820 nm for Alexa 594 and 880–900 nm for enhanced GFP. Two‐photon microscopes were custom‐built in house (Buchanan *et al*. 2012). The two‐photon microscope design was based on SliceScope (Scientifica Ltd), R3896 bialkali photomultipliers (Hamamatsu Corp., Bridgewater, NJ, USA) and 6215H 3‐mm (Cambridge Technologies, Bedford, MA, USA) or GVSM002/M 5‐mm (Thorlabs, Newton, NJ, USA) galvanometric mirrors. Ti:Sa laser gating was achieved using SH05/SC10 (Thorlabs) or Uniblitz LS6ZM2/VCM‐D1 (Vincent Associates, Rochester, NY, USA) shutters. Laser power was manually attenuated using a polarizing beam splitter (Thorlabs GL10‐B with AHWP05M‐980 half‐lambda plate) at the same time as monitoring output with a PM100A/S121C power meter (Thorlabs). Fluorescence was collected with an FF665 dichroic and an FF01‐680/SP‐25 emitter (Semrock Inc., Rochester, NY, USA). Red *vs*. green fluorescence was selected with a t565lpxr (Chroma, Bellow Falls, VT, USA) or a FF560‐Di01 dichroic beam mirror (Semrock), a ET630/75 m (Chroma) red emitter, and a ET525/50 m (Chroma) or a FF01‐525/45‐25 (Semrock) green emitter. Imaging data were acquired using customized variants of ScanImage, version 3.5–3.7 (Pologruto *et al*. 2003) running in Matlab (The MathWorks, Natick, MA, USA) via PCI‐6110 boards (National Instruments).

#### Morphological classification of cells

After recordings, morphologies were acquired with ScanImage. The preparation was scanned at a frame rate of 2 Hz (2 ms/line, 512 × 512 pixels) and three frames were averaged for each optical section. Neurons were manually reconstructed from two‐photon laser scanning microscopy (2PLSM) imaging stacks of Alexa‐594 fluorescence using Neuromantic (http://www.reading.ac.uk/neuromantic) as described previously (Blackman *et al*. 2014). Morphological reconstructions were carried out blinded to electrophysiology results. All students who carried out reconstructions were initially trained on the same separate set of 2PLSM stacks containing four reference cells to ensure that morphologies were reconstructed in a standardized manner. Boundaries of neocortical layers (Fig. 2 *B* and *D*) were identified in laser‐scanning Dodt‐contrast image stacks acquired simultaneously with the 3D 2PLSM fluorescence stacks. L5 was distinguished by the presence of prominent L5 PCs with large somata, L4 by a slightly darker granular band and L1 by a conspicuous absence of cell bodies. Morphologies were quantified using in‐house custom software (Buchanan *et al*. 2012) running in Igor Pro, version 6.37 (WaveMetrics Inc.), as described below.

To enable the creation of density maps (Fig. 2 *B*), morphologies were first rotated a small amount around the soma to ensure that the pial surface was in ‘up’ position; they were then centred on the L4/L5 boundary, after which the density map was calculated. Each compartment was represented by a two‐dimensional Gaussian with amplitude proportional to compartment length and a fixed sigma set to 25 μm. Maps were created by summing all Gaussians for each reconstruction, mirrored to create symmetry, normalizing to permit averaging across reconstructions, gamma corrected to improve visualization of weak densities, assigned a colour look‐up table depending on axonal or dendritic identity, and finally merged by the logical OR operation.

Convex hulls of individual reconstructions were constructed by two‐dimensionally projecting axonal and dendritic arbours separately and then applying a Jarvis walk to each projection. Ensemble convex hulls (Fig. 2 *B*) are convex hulls of all convex hulls, including mirror‐image convex hulls, which enable comparison of ensemble hulls with density maps.

For the Sholl analysis (Fig. 2 *C*), reconstructions were first re‐centred on their somata and converted to radial co‐ordinates. In 6.5 μm steps, the number of compartments straddling circles of increasing radii was counted (Sholl, 1953). Ensemble Sholl diagrams were averaged without normalization.

BCs were clustered automatically and independently into type 1 and type 2 (Fig. 3) using agglomerative single‐linkage hierarchical clustering software custom‐made in Igor Pro, with the squared Euclidian distance as linkage metric. BCs were clustered based on the percentage amount of the axon convex hull that was above the boundary between L2/3 and L4. We used this measure because it provided a degree of normalization across reconstructions manually traced by different people; some individuals added a lot of detail, whereas others did not, and this measure was robust in the face of such variability. We used a 25% best‐cut selection criterion to assess the number of clusters (Everitt *et al*. 2011). The Igor Pro built‐in fuzzy *c*‐means clustering algorithm pre‐set to find *c* = 2 clusters classified BCs exactly the same way.

#### AMPA uncaging

NPEC‐AMPA dissolved in aCSF (1 mm) supplemented with 0.2 μm TTX‐citrate and 20 mm Hepes was locally puffed using a patch pipette (4–6 MΩ). Photolysis was achieved with a violet TTL‐gated solid‐state laser (405 nm, 150 mW, MonoPower‐405‐150‐MM‐TEC; Alphalas GmbH, Göttingen, Germany). Photomultipliers were protected from the violet laser using a BLP01‐488R‐25 long‐pass filter (Semrock). The violet laser was always at maximum power because this setting gave the most reproducible pulses in separate laser tests. Power was instead attenuated with a polarizing beam splitter (Thorlabs WPMH05M‐405 and GL10‐A); laser power at the objective back aperture was measured to ∼8 mW with a PM100A/S121C power meter (Thorlabs). A single pulse of 0.1–2 ms was used to release AMPA during each voltage step (–100 mV to +50 mV); the slow photorelease of AMPA is a property of the NPEC cage (Palma‐Cerda *et al*. 2012) (Table 1). During uncaging, the laser beam was focused at a dendritic location approximately 50 μm from the soma but, because NPEC‐AMPA photolyses so slowly, AMPA cannot be assumed to have been localized to this spot. Each voltage step lasted 6–10 s and the interstep interval was 15 s. To assess the effect of Naspm bath application, cells were held at –80 mV with uncaging of AMPA every 15 s. In separate experiments, the AMPAR‐specific blocker 2,3‐dihydroxy‐6‐nitro‐7‐sulphamoyl‐benzo[f]quinoxaline‐2,3‐dione (NBQX) was bath‐applied (10 μm) to verify that uncaging‐evoked currents were attributable solely to AMPAR activation. We verified that series resistance did not systematically vary across cell type (PCs: 28 ± 3 MΩ, *n* = 4; BCs: 24 ± 0.6 MΩ, *n* = 19; 25 ± 1 MΩ, *n* = 10; ANOVA, *P* < 0.05, although none of the pairwise comparisons was significant; *P*_PC_ *_vs._*_BC_ = 0.076; *P*_PC_ *_vs._*_MC_ = 0.18; *P*_BC_ *_vs._*_MC_ = 0.52; BCs with and without internal spermine were pooled because they were indistinguishable; with spermine: 25 ± 1 MΩ, *n* = 11; without spermine: 22 ± 0.5 MΩ, *n* = 8; *P* = 0.055). In experiments where we measured the kinetics of uncaging‐evoked responses (Fig. 9 *B*), we verified that we did not have spurious differences in animal age or perfusion temperature that could potentially explain the findings (age in postnatal days, PCs: 13 ± 0.4; BCs: 14 ± 0.3; MCs: 13 ± 0.2; ANOVA, *P* = 0.62; perfusion temperature, PCs: 32 ± 0.04°C; BCs: 32 ± 0.05°C; MCs: 32 ± 0.04; ANOVA, *P* = 0.32) (Fig. 9 *B*). Laser pulse durations were also indistinguishable (PCs: 1.0 ± 0.1 ms; BCs: 1.1 ± 0.1 ms; MCs: 1 ± 0.1 ms, Kruskal–Wallis, *P* = 0.5), as were the uncaging‐evoked response amplitudes (PCs: –53 ± 9 pA; BCs: –60 ± 8 pA; MCs: –77 ± 11 pA, ANOVA, *P* = 0.3), suggesting that accidental differences in uncaging pulse properties could not explain our findings.

#### Computer modelling

A BC was modelled as a simplistic leaky integrate‐and‐fire point neuron with a single double‐exponential synaptic input (Dayan & Abbott, 2001). Differential equations were numerically integrated in Igor Pro, version 6.37 (WaveMetrics Inc.) using the forward Euler method with a time step of Δ*t* = 0.1 ms. The model was hand‐tuned approximately to biologically measured parameters (Tables 1 and 3), with membrane time constant τ_M_ = 10 ms, cell reversal potential *E*_leak_ = 70 mV, input resistance *R*_in_ = 160 MΩ, cell capacitance *C*_M_ = 62.5 pF (implicit from τ_M_ = *R*_in_ *C*_M_), AP threshold *V*_thresh_ = –37 mV, AP amplitude *V*_peak_ = 20 mV, AP reset voltage *V*_reset_ = –80 mV, synaptic reversal potential *E*_rev_ = 0 mV and synaptic conductance double‐exponential fast time constant τ_rise_ = 1.4 ms. The synaptic conductance double exponential slow time constant was set to τ_decay_ = 3 ms to simulate a fast‐decaying CP‐AMPAR‐mediated synaptic input, or to τ_decay_ = 5 ms to simulate an excitatory input of MC type but in a cell body with BC intrinsic properties. The goal of this model was thus not biological realism. Rather, because BCs and MCs also vary with respect to, for example, membrane time constant (Buchanan *et al*. 2012), our modelling approach aimed to tease apart the specific contribution of rapid AMPAR kinetics to BC response properties, in the absence of other contributing factors such as filtering by τ_M_ and short‐term plasticity.

We defined the first and second rheobase conductance values, *g*_rheo1_ and *g*_rheo2_, as the lowest peak synaptic conductances, *g*_syn_, for which one and two spikes, respectively, were obtained. This approximated a condition in which multiple excitatory inputs co‐operate to bring a postsynaptic BC just beyond threshold for one and for two APs, which had the additional advantage of providing normalization to enable comparison with conductance clamp experiments (see ‘Dynamic clamp’ above). We determined *g*_rheo1_ and *g*_rheo2_ using a bisection algorithm iterated 15 times. This approach established first and second rheobase conductance values to the second decimal place. For fast CP‐AMPAR‐style synaptic decay kinetics typical of excitatory synapses onto BCs (τ_decay_ = 3), we obtained *g*_rheo1_ = 13.03 nS and *g*_rheo2_ = 22.09 nS. For relatively slow synaptic decay kinetics characteristic of excitatory inputs to MCs (τ_decay_ = 5), *g*_rheo1_ = 11.15 nS and *g*_rheo2_ = 17.76 nS.

### Statistical analysis

The results are reported as the mean ± SEM. Significance levels are denoted using asterisks (*P < 0.05, **P < 0.01 and ***P < 0.001). Boxplots indicate the median value (middle black line), the 25th and 75th percentiles (box), and the highest and lowest values (whiskers), with the black cross denoting the mean.

Unless otherwise stated, we used Student's *t* test for equal means for all pairwise comparisons. If an equality of variances *F* test gave *P* < 0.05, we employed the unequal variances *t* test. Individual data sets were tested using a one‐sample *t* test. For multiple comparisons, pairwise comparisons were carried out if one‐way ANOVA suggested this at the *P* < 0.05 significance level. Equal or unequal variances (Welch) ANOVA was used depending on Bartlett's test for equal variances. For data that were not normally distributed, we used the Kruskal–Wallis test, as stated. Multiple pairwise comparisons were corrected *post hoc* using the method of Bonferroni–Dunn. Non‐parametric tests were always used in parallel with parametric tests, and were in agreement with respect to significance, although, occasionally, they gave a different significance level. Statistical tests were performed in Igor Pro, version 6.37 (WaveMetrics Inc.).

## Results

### Differential GluA2 labelling of Pvalb and Sst‐expressing INs

**Figure 1:**
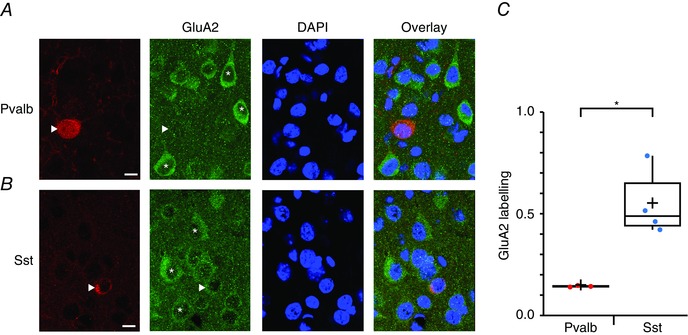
**Lower GluA2 immunolabelling in Pvalb‐positive than in Sst‐positive INs** *A*, confocal images of coronal sections of L5 visual cortex from a P21 WT mouse stained with DAPI and immunolabelled with antibodies against Pvalb and GluA2 (single optical slice). The image furthest to the right is a composite of the three to its left. The Pvalb‐positive IN (arrowhead) lacks GluA2 labelling. Asterisks indicate PCs labelled for GluA2. Scale bar = 10 μm. *B*, as in (*A*), but for an Sst‐positive IN in an acute slice from another P21 WT mouse. The Sst‐positive IN (arrowhead) is positively labelled for GluA2, although less so than in nearby PCs (asterisks). *C*, boxplot quantifying the considerably lower GluA2 expression in Pvalb‐positive compared to Sst‐positive INs. IN GluA2 labelling was quantified as the fluorescence intensity less background with PC GluA2 labelling as a normalizing reference (see Methods). For Pvalb‐positive cells, data were from three mice (nine stacks; 156 background ROIs, 171 PCs and 116 Sst‐positive INs). For Sst‐positive cells, data were from four mice (12 stacks;146 background ROIs, 145 PCs and 61 Sst‐positive INs).

To assess the relative expression of GluA2 in BCs and MCs within L5 of the mouse visual cortex, we first examined the pattern of GluA2 immunoreactivity in these INs, identified by the presence of Pvalb and Sst, respectively. In slices from P21 WT mice, we quantified immunolabelling in neocortical L5, which was identified by the presence of large PC somata. Unsurprisingly, L5 PCs were immunoreactive for GluA2 (Kumar *et al*. 2002). Although Pvalb‐positive somata showed little somatic immunolabelling for GluA2 (Fig. 1 *A*), Sst‐positive somata were strongly labelled but less so than those of PCs (Fig. 1 *B*). We quantified somatic GluA2 fluorescence by normalizing to that of PCs, which indicated a much greater GluA2 labelling of Sst‐positive cells than of Pvalb‐positive neurons (normalized intensity 0.55 ± 0.08 for Sst *vs*. 0.14 ± 0.002 for Pvalb; *n* = 3 and 4, *P* < 0.05, Student's *t* test; see Methods) (Fig. 1 *C*). This suggests a differential expression of CP‐AMPARs in L5 BCs and MCs.

### Morphological classification of recorded INs identified MCs and two types of BCs

To examine whether the cell‐dependent expression of GluA2 was reflected in the properties of synaptic currents, we performed a series of experiments using whole‐cell recording (see below). This required reliable targeting and classification of recorded cells.

L5 PCs were readily targeted using contrast‐enhanced infrared video microscopy because of their large pyramidal somata and conspicuous apical dendrites. BCs were similarly targeted by their relatively small and rounded cell bodies or by fluorescence in acute slices from the Pvalb‐positive G42 mouse line (Chattopadhyaya *et al*. 2004), visualized by 2PLSM. To target MCs by fluorescence, we used the Sst‐positive GIN transgenic mouse line (Oliva *et al*. 2000), which solely labels MCs in neocortical L5 (Fino & Yuste, 2011; Buchanan *et al*. 2012). Alternatively, MCs were targeted in slices from WT mice by their large and characteristically ovoid somata (Silberberg & Markram, 2007).

**Figure 2:**
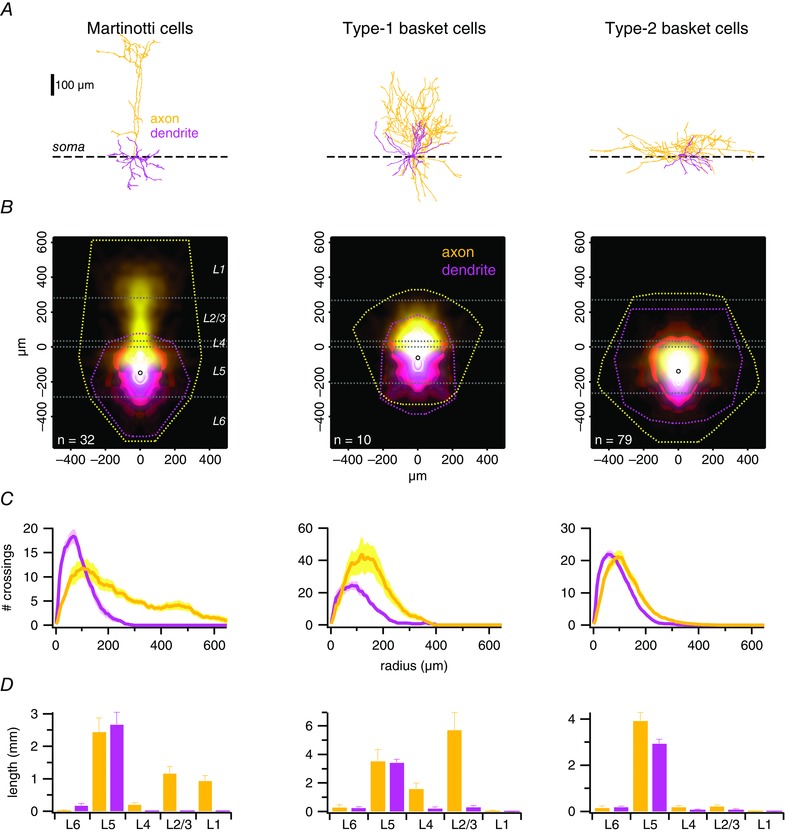
**Laminar distribution of axon identified MCs and two types of BCs** *A*, sample MC, type‐1 and type‐2 BC morphologies, aligned on their somata (dashed line). *B*, ensemble density maps (see Methods) (Buchanan *et al*. 2012) of all recorded INs show typical axonal (yellow) and dendritic (magenta) arborizations. Convex hulls (dashed lines) illustrate maximum axonal and dendritic extents. Open circles indicate the average position of cell bodies. Horizontal white dashed lines denote the neocortical layer boundaries averaged across cells. *C*, ensemble Sholl diagrams show the number of axonal (yellow) or dendritic (pink) branches crossing a given radial distance from the soma (Sholl, 1953). The prominent ascending axon of type‐1 BCs distinguishes them from the classical type‐2 BCs, whose peak axonal radial density is closer to the soma (Buchanan *et al*. 2012; Ferreira *et al*. 2014). *D*, total length of axonal arbours (yellow) within a neocortical layer distinguished different cell types well, whereas the dendritic branching pattern (magenta) was less useful for classification. Axons of type‐1 but not of type‐2 BCs branched extensively in L2/3 but never reached L1. MC axons, however, consistently reached L1.

Every recorded neuron was classified *post hoc* by morphology (Fig. 2 and Table 2). Recorded INs were manually reconstructed from 2PLSM imaging stacks and morphological classification was carried out (see Methods) (Buchanan *et al*. 2012; Blackman *et al*. 2014). MCs were readily distinguished from BCs by virtue of their characteristic ascending axon that ramified in L2/3 and extended into L1, in combination with dendrites descending below the soma (Fig. 2) (Buchanan *et al*. 2012).

**Table 2:** **All INs were morphologically identified**

Experiment	BC (*n*)	MC (*n*)
Rectification, pairs	14	10
Rectification, NPEC‐AMPA uncaging, with spermine	11	10
Rectification, NPEC‐AMPA uncaging, no spermine	8	NA
Naspm wash‐in, pairs	6	NA
Naspm wash‐in, pairs (control)	6	NA
Naspm wash‐in, NPEC‐AMPA uncaging	7	7
Naspm wash‐in, NPEC‐AMPA uncaging (control)	7	3
NBQX wash‐in, NPEC‐AMPA uncaging	2	2
Rectification of mEPSCs, no spermine	9	NA
Rectification of mEPSCs, with spermine	12	NA
Naspm wash‐in, mEPSCs	5	NA
Naspm wash‐in, mEPSCs (control)	3	NA
Dynamic clamp	5	NA
Total	95	32

Morphologies in **Fig**. 2 were obtained from 89 BCs and 32 MCs, which constitutes the entire IN data set of the present study. Paired recordings in **Fig**. 5 included data from two triplet recordings, for which two PCs were connected to the same postsynaptic BC. Additionally, four PC‐BC connections used in rectification measurement (**Fig**. 5***D*** and ***E***) also served as stability controls for Naspm wash‐in experiments (**Fig**. 5***G*** and ***H***). Together, this results in a total of 95 experiments in BCs, even though the total number of reconstructed BC morphologies is 89. NA, not available.

**Figure 3:**
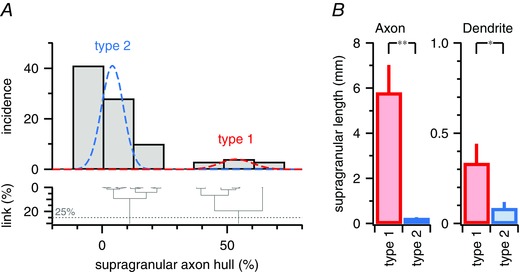
**Axonal morphology classified BCs into two types** *A*, BC morphologies were independently clustered based on the amount axonal branching in supragranular layers (see Methods). The 25% best‐cut (dotted line) intersected the dendrogram (bottom) twice, suggesting that BCs should be partitioned into two types. Type 1 (red) had ascending axons ramifying extensively above the border of granular L4, whereas the axonal arbour of type 2 (blue) was largely subgranular (Fig. 2) (Buchanan *et al*. 2012; Ferreira *et al*. 2014). *B*, total supragranular axon length was considerably different for type‐1 and type‐2 BCs (5.8 ± 1 mm, *n* = 10, *vs*. 0.23 ± 0.05 mm, *n* = 71, *P* < 0.01). We also found a small but significant difference in total supragranular dendrite length (330 ± 100 μm *vs*. 90 ± 40 μm, *P* < 0.05).

BCs were additionally independently morphologically classified into two subtypes using software clustering (see Methods) (Fig. 3). Axons of type‐1 BCs preferentially ramified in L2/3, whereas axons of type 2 branched chiefly in L5, as reported previously (Buchanan *et al*. 2012; Ferreira *et al*. 2014). The type‐1 and type‐2 BCs were found at indistinguishable rates in slices from Pvalb‐positive G42 (three out of 11) and WT mice (seven out of 78, *P* = 0.072, chi‐squared test).

**Figure 4:**
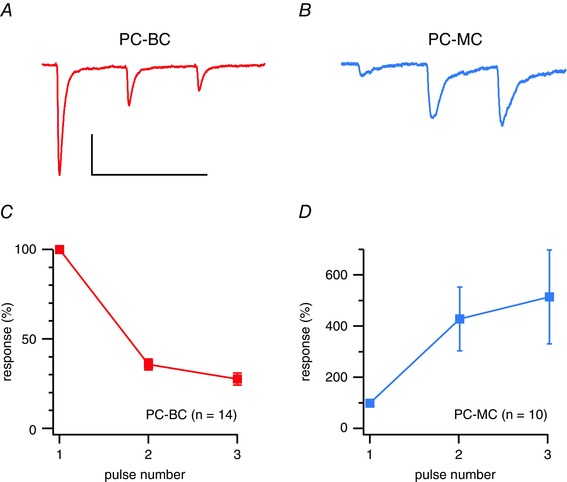
**PC‐BC connections were short‐term depressing, whereas PC‐MC synapses facilitated** *A*, sample voltage‐clamp trace illustrates a PC‐BC connection that characteristically exhibits short‐term depression (Blackman *et al*. 2013). Three APs were repeatedly elicited at 30 Hz in the presynaptic PC and 20 postsynaptic sweeps were averaged every 15 s. Scale bars = 50 ms, 50 pA. *B*, PC‐MC connection recorded under the same conditions shows typical short‐term facilitation (Blackman *et al*. 2013). *C*, ensemble average shows that PC‐BC short‐term depression is robust. Responses were normalized to the first in a train. *D*, in contrast, PC‐MC synapses strongly and robustly facilitated. Short‐term plasticity properties could thus be used to distinguish MCs from BCs.

Whenever possible, cell type was additionally verified by synaptic response dynamics (Fig. 4 and Table 1). Excitatory inputs to BCs characteristically showed short‐term depression, whereas excitatory connections to MCs exhibited strong short‐term facilitation (Buchanan *et al*. 2012; Blackman *et al*. 2013). In a subset of recordings, BCs were also identified by their characteristic high‐threshold fast‐spiking pattern (Table 3) (Buchanan *et al*. 2012).

**Table 3:** **Intrinsic properties of BCs**

Parameter	Value
Spike threshold (mV)	–33 ± 1
Spike height (mV)	55 ± 3
Spike half‐width (ms)	0.63 ± 0.07
Spike after‐hyperpolarization (mV)	–19 ± 1
Rheobase current (pA)	220 ± 30
Frequency (Hz)	55 ± 10
Accommodation (%)	–5.6 ± 10
Coefficient of variation (%)	8.0 ± 2
Membrane potential, *V*_M_ (mV)	–70 ± 2
Input resistance, *R*_in_ (MΩ)	150 ± 10
Membrane time constant, τ_M_ (ms)	11 ± 2

Data obtained from BCs in **Fig**. 8***D*** to ***F***.

### Synaptic currents suggest the presence of CP‐AMPARs at PC‐BC but not at PC‐MC or at PC‐PC connections

**Figure 5:**
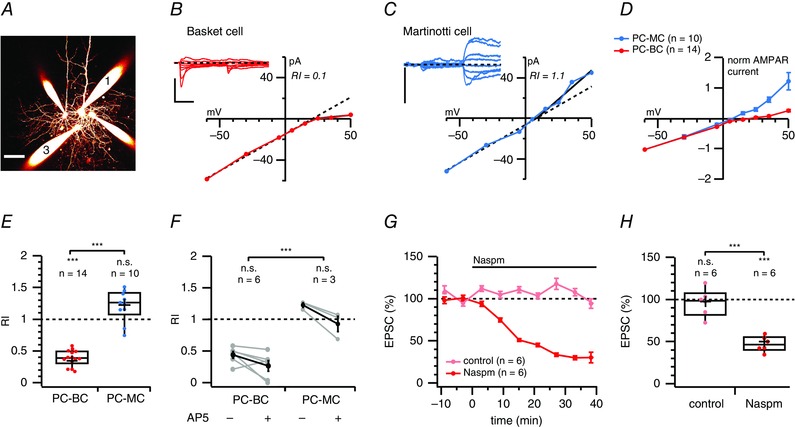
**Monosynaptic connections from PCs to BCs but not to MCs rectify** *A*, 2PLSM maximum intensity projection of a quadruple whole‐cell recording in which cell 1 was a PC connected to cell 3, a BC. These cells were identified by morphology and electrophysiology (see Methods). Scale bar = 50 μm. *B*, two APs evoked at 30 Hz in PC1 gave rise to short‐term depressing synaptic responses in BC3 (inset, average of 10 traces) that rectified at positive membrane potentials. The RI_slope_ (0.1) was calculated as the *I*–*V* slope for voltages more positive than the reversal potential divided by the slope at more negative potentials (see Methods). Dashed diagonal line denotes the expected *I*–*V* relationship in the absence of rectification. Scale bars = 10 ms, 100 pA. *C*, as in (*B*), but for a PC‐MC connection without inward rectification. As a result of the low initial probability of release, the second EPSC was analysed. Scale bar = 50 pA. *D*, normalized and averaged *I*–*V* curves of PC‐BC connections (red) and PC‐MC connections (blue) indicated that this difference in rectification was specific to synapse type and not a random heterogeneity. *E*, PC‐BC synapses (red) were inward rectifying, whereas PC‐MC synapses (blue) were not (*P* = 0.35). The RI of PC‐BC pairs was in addition different from that of PC‐MC connections (Bonferroni corrected). RI values in cells recorded from GIN and WT mice were indistinguishable (0.90 ± 0.06, *n* = 4 *vs*. 1.2 ± 0.2, *n* = 6, *P* = 0.12). *F*, in a subset of recordings, we examined whether NMDAR currents biased our CP‐AMPAR rectification measurements but found that AP5 wash‐in had no effect on RI measurements at PC‐BC or PC‐MC connections (paired two‐sample *t* tests). As in (*E*), the difference in RI between PC‐MC and PC‐BC connections was significant. *G*, ensemble averages show the time course of Naspm blockade of PC‐BC EPSC (red) compared to stable mock wash‐in controls (light red). *H*, mock wash‐in controls were stable (*P* = 0.81 *vs*. 100%), whereas Naspm halved the PC‐BC EPSC amplitude, implying the presence of CP‐AMPARs at this connection type.

We first performed whole‐cell recordings of connected PC‐IN and PC‐PC pairs (Fig. 5 *A*). Once a connection was found, the postsynaptic IN was held at different membrane potentials ranging from –100 mV to +50 mV during which APs were evoked in the presynaptic PC. Recordings were performed using an intracellular solution containing added spermine (see Methods). For PC‐BC connections, the *I*–*V* relationship was inwardly rectifying (Fig. 5 *A*, *B* and *D*), as indicated by a low RI (Fig. 5 *E*), reflecting the predominance of CP‐AMPARs. By contrast, the *I*–*V* relationship of PC‐MC connected pairs was linear (Fig. 5 *C* to *E*), suggesting a predominance of CI‐AMPARs. At PC‐PC connections, synaptic currents exhibited a non‐rectifying *I*–*V* relationship (RI_slope = _1.1 ± 0.1, *n* = 4 pairs, *P* = 0.69 for comparison with 1; data not shown), which was different from PC‐BC pairs (*P* < 0.001, Bonferroni adjusted). The RIs of PC‐PC and PC‐MC connections were indistinguishable (*P* = 0.75).

Although we analysed the initial 1–3 ms of EPSCs, we were concerned that NMDARs might contaminate the analysis. We therefore blocked NMDARs with AP5, although RI at both PC‐BC and PC‐MC synapses was unaffected (Fig. 5 *F*). Taken together, these results suggest the expression of CP‐AMPARs at PC‐BC but not PC‐MC or PC‐PC synapses.

We proposed that, if CP‐AMPARs are indeed present at PC‐BC synapses, the blocker Naspm (Koike *et al*. 1997) should decrease the amplitude of PC‐BC EPSCs. Consistent with this prediction, we found that, after wash‐in of 200 μm Naspm, PC‐BC EPSCs were reduced, whereas control recordings remained stable (Fig. 5 *G* and *H*), confirming the presence of CP‐AMPARs. The absence of complete blockade suggests the possible combined expression of CI and CP‐AMPARs at PC‐BC synapses or, alternatively, that Naspm incompletely blocks CP‐AMPARs (see ‘Discussion’).

For PC‐MC connections, Naspm wash‐in experiments reduced neurotransmission (data not shown). CP kainate receptors are expressed presynaptically at excitatory inputs to Sst‐positive INs in the hippocampus, and can be blocked by Naspm (Sun *et al*. 2009). Such receptors may also be present presynaptically at inputs to the corresponding neocortical Sst‐positive Ins (i.e. MCs). Because a presynaptic effect of Naspm would make it impossible to assess thhe postsynaptic impact of Naspm, this approach could not be used to determine the presence or absence of CP‐AMPARs at PC‐MC connections. As a solution, we switched from paired recordings to AMPA uncaging (see below).

### Both BCs and MCs possess postsynaptic NMDA receptors

**Figure 6:**
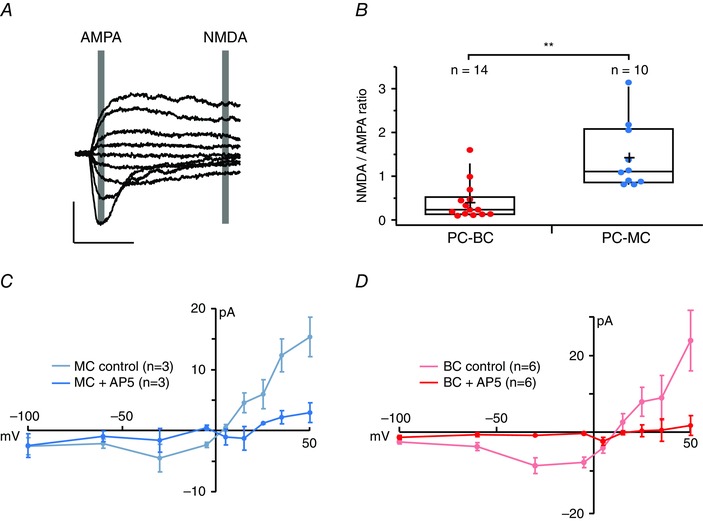
**Both PC‐MC and PC‐BC connections have postsynaptic NMDARs** *A*, AMPAR and NMDAR‐mediated currents were measured at latencies indicated by grey boxes (see Methods). These sample sweeps are from a PC‐MC paired recording. Scale bars = 10 ms, 100 pA. *B*, PC‐BC connections (red) had smaller NMDA/AMPA ratios than PC‐PC (black) or PC‐MC connections (blue), which might appear to suggest that the former have fewer postsynaptic NMDARs. NMDA/AMPA ratios in cells recorded from GIN and WT mice were indistinguishable (0.9 ± 0.06, *n* = 4 *vs*. 1.4 ± 0.2, *n* = 6, *P* = 0.08). *C*, in agreement with the existence of postsynaptic NMDARs at PC‐MC connections, an *I*–*V* relationship characteristic of an NMDAR‐mediated current was found in the absence (blue) but not the presence of the NMDAR antagonist AP5 (light blue). *D*, evidence for postsynaptic NMDARs was found at PC‐BC connections as well: an *I*–*V* relationship characteristic of the NMDAR‐mediated current was found in the absence (red) but not the presence of the NMDAR antagonist AP5 (pink).

As an aside, the NMDA_+50_/AMPA_–60_ ratio was 3‐fold smaller at PC‐BC than at PC‐MC connections (Fig. 6 *A* and *B*). Initially, this suggested that PC‐MC synapses expressed more NMDARs relative to AMPARs or had a higher NMDAR conductance than did PC‐BC synapses. However, this difference could also be explained by a differential space‐clamp error in BCs and MCs. Indeed, long‐latency synaptic currents at PC‐MC, as well as at PC‐BC connections, had an *I*–*V* relationship characteristic of NMDARs that was abolished by AP5 (Fig. 6 *C* and *D*), suggesting that both these synapse types do possess postsynaptic NMDARs.

### Currents evoked by AMPA uncaging rectify in BCs but not in MCs or PCs

**Figure 7:**
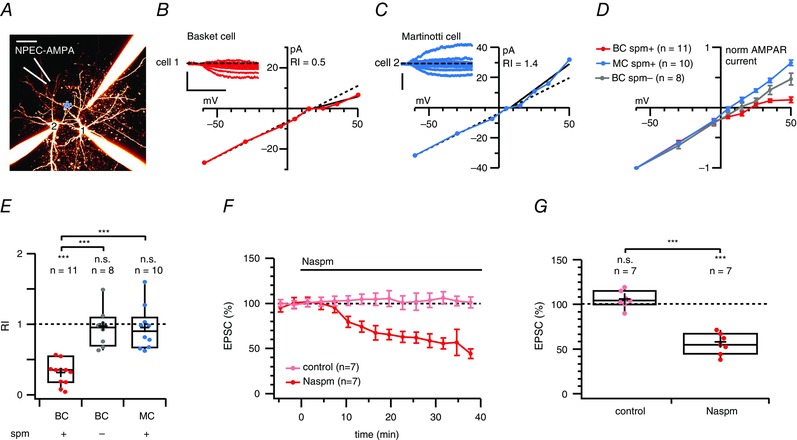
**AMPA uncaging currents rectify in BCs but not in MCs** *A*, 2PLSM maximum intensity projection of a triplet IN recording, with a fourth pipette (white) used for puffing NPEC‐AMPA. Cells were morphologically identified as BC (cell 1) and MC (cell 2) cells. A blue asterisk indicates the location of the 405 nm laser spot. Scale bar = 50 μm. *B*, submillisecond 405 nm laser pulses elicited AMPAR responses in the BC (cell 1, inset) with slow kinetics (as expected from the NPEC cage) (Palma‐Cerda *et al*. 2012) that rectified at positive membrane potentials, suggesting the presence of CP‐AMPARs. The RI_slope_ (0.3) was calculated as the *I*–*V* slope of peak photolysis‐evoked currents at voltages more positive than the reversal potential divided by the slope at more negative potentials (see Methods). Scale bars = 500 ms, 25 pA. *C*, for the MC recorded in parallel, however, AMPA uncaging responses did not rectify (cell #2, inset). Scale bars = 150 pA. *D*, normalized and averaged *I*–*V* curves of AMPA uncaging responses recorded in BCs (red) and in MCs (blue) showed that this difference in inward rectification was specific to cell type. In control experiments without internal spermine, BCs did not rectify (grey). *E*, NPEC‐AMPA photolysis‐evoked responses were rectifying in BCs (red) but not in MCs (blue, *P* = 0.1), nor in BCs without internal spermine (grey, *P* = 0.6). In addition, the RI measured in BCs was different from that in MCs and in BCs without internal spermine (Bonferroni corrected), whereas the RIs of MCs and BCs without internal spermine were indistinguishable. The RI in cells recorded from GIN and WT mice was also indistinguishable (0.90 ± 0.1, *n* = 6 *vs*. 0.92 ± 0.1, *n* = 4, *P* = 0.91). *F*, ensemble averages show the time course of Naspm blockade of AMPA‐uncaging‐evoked responses (red) compared to stable mock wash‐in controls (light red). *G*, although mock wash‐in controls were unaffected (*P* = 0.21), Naspm decreased NPEC‐AMPA photolysis‐evoked responses by half, suggesting the widespread presence of CP‐AMPARs in this cell type.

To eliminate any contribution from presynaptic kainate receptors (Sun *et al*. 2009) and to focus exclusively on the postsynaptic side, we uncaged AMPA by photolysing NPEC‐AMPA with brief 405 nm laser pulses. We used aCSF supplemented with 0.2 μm TTX to exclude a possible contribution of glutamate as a result of the suprathreshold activation of neighbouring cells. We puffed NPEC‐AMPA close to the soma and proximal dendrites of PCs, BCs or MCs using a patch pipette. Recorded neurons were voltage‐clamped at potentials ranging from –100 mV to +50 mV and a single 0.1–2 ms laser pulse per voltage step produced a slow current (Fig. 7 *A* and *B*) characteristic of the slow rate of photolysis of the NPEC cage (Palma‐Cerda *et al*. 2012). The uncaging‐evoked current was almost abolished by 10 μm NBQX (7% ± 2% of baseline, *n* = 4 cells, *P* < 0.001 compared to 100%; data not shown), indicating that it was indeed AMPAR‐mediated. With AMPA uncaging, we found a marked rectification in BCs (Fig. 7 *D* and *E*). By contrast, no such rectification was observed in MCs with internal spermine, BCs without internal spermine (Fig. 7 *D* and *E*) or in PCs with internal spermine (RI_slope_ = 0.9 ± 0.1, *n* = 5 cells, *vs*. 1, *P* = 0.31; data not shown). These results are in keeping with the paired recording experiments and confirm the presence of CP‐AMPARs in BCs.

We next examined the effect of Naspm on currents evoked by AMPA uncaging in BCs held at –80 mV. Naspm wash‐in decreased the amplitude of uncaging‐evoked responses, whereas control recordings remained stable (Fig. 7 *F* and *G*). By contrast, Naspm wash‐in did not affect AMPA‐uncaging‐evoked currents in PCs (94% ± 5% of baseline, *n* = 6 cells, *vs*. 100%, *P* = 0.29; data not shown) or MCs (95% ± 4%, *n* = 7 cells, *vs*. 100%, *P* = 0.18; or *P* = 0.08 *vs*. MC mock Naspm controls 103% ± 2%, *n* = 3; data not shown). These uncaging experiments corroborate the paired‐recording results and show that CP‐AMPARs are specifically expressed in neocortical L5 BCs but not MCs or PCs. Because NPEC‐AMPA uncaging activates synaptic and extrasynaptic AMPARs, these experiments further suggest that CP‐AMPARs may be selectively expressed in BCs in a cell‐wide manner.

### CP‐AMPARs contribute to miniature EPSCs in BCs

Because paired recordings sample a small fraction of all synaptic inputs onto a cell, it is possible that we missed excitatory inputs onto BCs that do not contain CP‐AMPARs. Spontaneous release, however, can arise at any of the synaptic contacts onto a neuron and dendritic filtering should not reduce the chances of detecting spontaneous release events at distal synapses of relatively electrically compact BCs (Sjöström *et al*. 2008; Maheux *et al*. 2015). Spontaneous release may thus sample relatively globally from all excitatory inputs onto a recorded cell. Moreover, spontaneous and evoked glutamate release may activate non‐overlapping populations of receptors and synapses (Atasoy *et al*. 2008; Sutton & Schuman, 2009; Sara *et al*. 2011; Peled *et al*. 2014). To determine whether spontaneously released glutamate activates AMPARs with functional properties similar to those activated in an AP‐dependent fashion, we examined the contribution of CP‐AMPARs to mEPSCs recorded in BCs.

**Figure 8:**
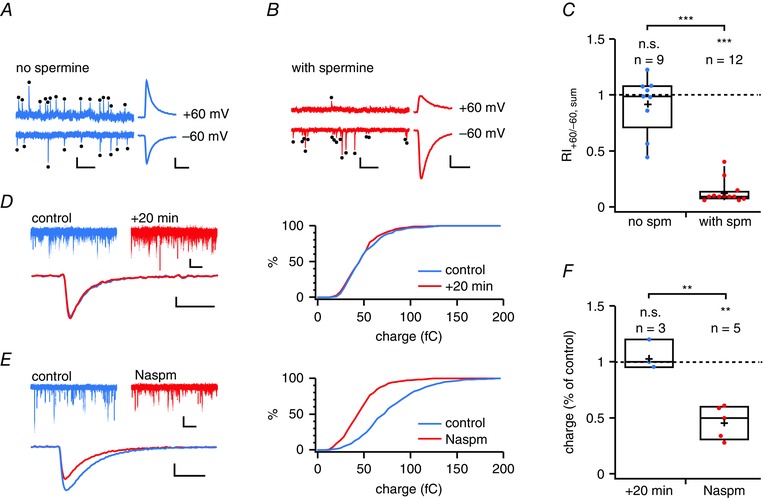
**Rectification of BC mEPSCs is spermine‐dependent** *A*, representative BC mEPSCs (black dots) recorded at –60 mV and +60 mV using spermine‐free internal solution. Scale bars = 100 ms, 50 pA. Right: average of all mEPSCs at +60 mV and count‐matched average at –60 mV are of similar absolute amplitudes, suggesting an absence of rectification. Scale bars = 2 ms, 20 pA. *B*, as in (*A*) but for a BC recorded with internal solution supplemented with spermine. Right: average of all mEPSCs at +60 mV is of smaller absolute amplitude than the count‐matched average at –60 mV, indicating rectification. Scale bars as in (*A*). *C*, BC mEPSC rectified in the presence (red) but not the absence of spermine (blue, *P* = 0.37 for the comparison with 1), suggesting CP‐AMPAR‐mediated quantal currents. The RI of mEPSCs was different from that without spermine. The rectification index, RI+60/–60, was calculated from the summed charge (see Methods). Half of the cells were targeted in the Pvalb‐positive G42 mouse line (6/12 for ‘with spm’ and 5/9 for ‘no spm’) (Chattopadhyaya *et al*. 2004). *D*, representative control recording at –60 mV showing that the mEPSC charge remained stable for 20 min (*P* = 0.95 using the Kolmogorov–Smirnov test). Scale bars: top = 500 ms, 20 pA; bottom = 2 ms, 10 pA. *E*, Naspm wash‐in reduced the mEPSC charge (*P* < 0.01, Kolmogorov–Smirnov), suggesting the presence of CP‐AMPARs. Scale bars as in (*D*). *F*, mEPSC summed charge was unaffected by mock wash‐in (*P* = 0.56 for comparison with 100%), whereas bath application of Naspm approximately halved the mEPSC summed charge, indicating the cell‐wide presence of CP‐AMPARs in BCs. All cells were WT. Mini frequency (12 ± 3 Hz) was typical of BCs (Buchanan *et al*. 2012).

To isolate AMPAR‐mediated spontaneous currents, we blocked voltage gated Na^+^ channels, NMDARs, GABA_A_ receptors and GABA_B_ receptors using TTX, AP5, SR‐95531 and CGP 54626 (see Methods). We measured mEPSC rectification RI_+60/–60_ in BCs with and without added intracellular spermine (Bats *et al*. 2012). This revealed spermine‐dependent inward rectification, indicating the activation of synaptic CP‐AMPARs by quantal events in BCs (Fig. 8 *A* to *D*). To verify these findings pharmacologically, we recorded mEPSCs from BCs at –60 mV with washing in Naspm. In agreement with the rectification data, mEPSC‐mediated charge transfer was decreased by Naspm (Fig. 8 *E* to *G*). Taken together, our findings suggest that both spontaneous and evoked glutamate release activates CP‐AMPARs in BCs, implying a cell‐wide expression.

### EPSCs decay more rapidly in BCs than in MCs and PCs

**Figure 9:**
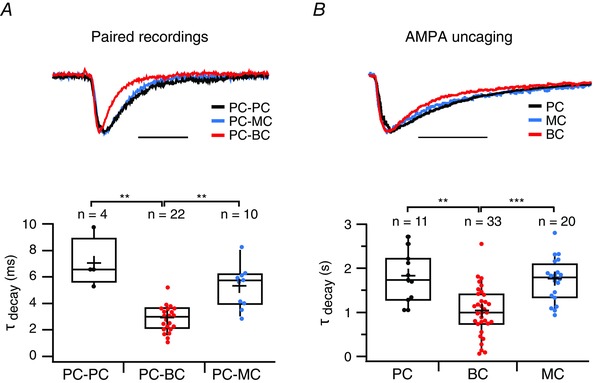
**AMPAR currents decay faster in BCs than in PCs and MCs** *A*, top: representative voltage‐clamp traces showing faster decay kinetics at a PC‐BC synapse (red) than at a PC‐PC (black) and a PC‐MC connection (blue). Scale bar = 10 ms. *A*, bottom: the decay time constant, τ_decay_, was faster for PC‐BC (red) than for PC‐PC (black) and PC‐MC (blue) connections. PC‐PC and PC‐MC connections were indistinguishable with respect to τ_decay_ (*P* = 0.06). PC‐BC synapses were measured at –100 mV or at –80 mV and, because the decay times at these two voltages were indistinguishable, these data were pooled (τ_decay,–100 mV_ = 2.8 ± 0.3 ms, *n* = 14 pairs and τ_decay,–80 mV_ = 3.2 ± 0.3 ms, *n* = 8 pairs, *P* = 0.22, data not shown). *B*, top: representative voltage‐clamp traces showing AMPA‐uncaging responses with faster decay kinetics in BCs (red) than in PCs (black) and MCs (blue). Scale bar = 2 s. *B*, bottom: the decay time constant, τ_decay_, was faster for AMPA uncaging responses in BCs (red) than in PCs (black) and MCs (blue). Data were acquired at either –100 mV or at –80 mV, and were pooled because the decay time constants at these two voltages were indistinguishable (BCs: τ_decay,–100 mV_ = 1.0 ± 0.1 s, *n* = 19 cells, τ_decay,–80 mV_ = 1.1 ± 0.1 s, *n* = 14 cells, *P* = 0.7; MCs: τ_decay,–100 mV_ = 1.8 ± 0.1 s, *n* = 10 cells, τ_decay,–80 mV_ = 1.2 ± 0.4 s, *n* = 10 cells, *P* = 0.2; PCs: τ_decay,–100 mV_ = 2.0 ± 0.3 s, *n* = 5 cells, τ_decay,–80 mV_ = 1.6 ± 0.2 s, *n* = 6 cells, *P* = 0.2; data not shown). For BCs, τ_decay, –100_ data were pooled for with and without internal spermine because these conditions were indistinguishable (τ_decay, –100, spm+ = _1.1 ± 0.2 s; *n* = 11; τ_decay, –100, spm– = _0.9 ± 0.2 s, *n* = 8; *P* = 0.5). Uncaging responses recorded in PCs and in MCs were indistinguishable with respect to τ_decay_ (*P* = 0.24).

Fast‐spiking INs in hippocampus and neocortical L2/3, 4 and 6 have previously been associated with AMPARs with relatively rapid decay kinetics (Hestrin, 1993; Geiger *et al*. 1995; Angulo *et al*. 1997; Geiger *et al*. 1997). Accordingly, we investigated whether the presence of CP‐AMPARs at PC‐BC connections in L5 was also associated with more rapid synaptic kinetics. Indeed, the decay time constant, τ_decay_, for PC‐BC EPSCs was faster than for PC‐MC or PC‐PC EPSCs (Fig. 9 *A* and Table 1). However, the EPSC rise time constants, τ_rise_, were indistinguishable (PC‐PC: 2.2 ± 0.8 ms; PC‐BC: 1.8 ± 0.3 ms; PC‐MC: 1.9 ± 0.4 ms; *n* = 5, 22 and 10 pairs, respectively; ANOVA, *P* = 0.89). We were concerned that this difference in decay time constant was an artefact arising from differential filtering in the different cell types because BCs have faster membrane time constant, τ_M_, than MCs and PCs (Buchanan *et al*. 2012). To rule out a contribution from differential membrane time constant filtering, we benefitted from the fact that NPEC‐AMPA photolysis is one order of magnitude slower than τ_M_ in any of these cells (Fig. 7 *B* and *C*) (Palma‐Cerda *et al*. 2012). In agreement with the paired recordings, AMPA‐uncaging‐evoked responses decayed faster in BCs than in MCs or PCs (Fig. 9 *B* and Table 1), suggesting that the difference in kinetics was a result of cell‐specific AMPAR characteristics rather than biophysical properties intrinsic to these three cell types.

### Rapid AMPAR kinetics reduces latency and duration of spiking responses in BCs

**Figure 10:**
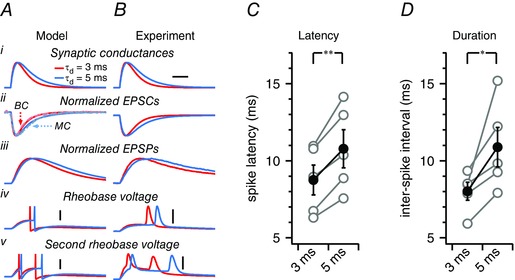
**Rapid AMPAR kinetics sharpens BC‐mediated inhibition** *A*, a leaky integrate‐and‐fire computer model was tuned to average BC intrinsic properties (see Methods). A single excitatory synaptic conductance (*Ai*) was modelled based on an excitatory input to a BC with rapid decay, τ_decay_ = 3 ms, or on an excitatory input to an MC with slow decay, τ_decay_ = 5 ms. For comparison, model EPSCs are represented together with representative EPSCs recorded in a BC and an MC (*Aii*, dashed traces). Even though the synaptic conductance rise time constant τ_rise_ was the same in both cases (Table 1), this gave rise to EPSPs with different rise times and peak latencies (*Aiii*) because of temporal integration. When the peak synaptic conductance amplitude was set to the lowest value at which one spike was evoked (‘rheobase’), fast‐decaying conductances consequently produced APs with shorter latencies than slow‐decaying conductances did (*Aiv*). To assess BC response duration, the peak synaptic conductance was increased to the lowest value at which two APs were evoked (denoted the ‘second rheobase’), which resulted in interspike intervals of shorter duration with the rapidly decaying input. Scale bars = 5 ms, 20 mV. *B*, to verify the computer model predictions, we carried out conductance clamp experiments (see Methods) because this allowed us to investigate the consequences of altered synaptic kinetics in a real BC. The same conductance kinetics was used as in the computer model (*Bi*), which again gave rise to EPSCs with fast and slow decay (*Bii*). The resulting EPSPs had different rise times and peak latencies (*Bii*). This resulted in a different AP latency (*Biv*) and response duration (*Bv*) in this particular BC, in agreement with the computer model. *C*, in dynamic clamp experiments, the AP latency was consistently shorter with rapidly decaying synaptic kinetics (paired *t* test). Grey circles denote measurements from individual BCs (*n* = 5) and black circles are the averages. *D*, response duration was also robustly shortened by rapidly decaying synaptic conductance (paired *t* test).

We explored the possible functional consequences of the faster synaptic kinetics in BCs by implementing a simple leaky integrate‐and‐fire computer model of a BC with a single synaptic input modelled as a double‐exponential conductance waveform. We used either τ_decay = _3 ms or τ_decay = _5 ms, which is consistent with the excitatory input kinetics seen in BCs and MCs, respectively (Fig. 10 *A*). The model predicted that rapidly decaying inputs result in EPSPs with faster rise, shorter spike latencies and temporally sharpened responses in BCs (Fig. 10 *B*). To test the model predictions, we carried out dynamic clamp experiments. We patched BCs and injected conductances mimicking rapidly or slowly decaying excitatory synaptic inputs (τ_decay = _3 ms or 5 ms), as employed for the computer model. In agreement with the model, in dynamic clamp, we observed more rapidly rising EPSPs (τ_rise_ = 3.5 ± 0.3 ms *vs*. 5.4 ± 0.8 ms, *n* = 5, *P* < 0.05, paired *t* test), shorter spike latencies and temporally sharpened responses for the rapidly decaying excitatory inputs (Fig. 10 *B* and *C*). Rapidly decaying EPSCs occurring at CP‐AMPAR‐expressing PC‐BC synapses may thus contribute to the rapid BC‐mediated early inhibition of PCs.

## Discussion

In the present study, we demonstrate a contrasting CP‐AMPAR expression pattern at excitatory synapses onto two major IN subclasses in L5 of visual cortex, with BCs but not MCs expressing CP‐AMPARs. Although the somata of Pvalb‐positive INs contained little GluA2 immunoreactivity, the somata of Sst‐positive INs were more strongly labelled but less so than PCs. This observation was supported by our electrophysiological data, which showed both rectifying *I*–*V* relationships and Naspm sensitivity for AMPAR‐mediated currents in BCs but no rectification in MCs or in PCs. In addition, CP‐AMPAR‐expressing synapses were associated with more rapidly decaying kinetics. Computer modelling predicted that this helps to shorten BC response latency and duration, which we verified using conductance clamp experiments.

### Interneuron classification

To ensure correct identification of recorded INs, we reconstructed and morphologically characterized all INs. Whenever possible, we also classified them based on firing pattern and short‐term plasticity of excitatory inputs. In addition, in a subset of recordings, we used two transgenic mouse lines that indicate cells are positive for Pvalb or for Sst by fluorescence labelling (Oliva *et al*. 2000; Chattopadhyaya *et al*. 2004). As expected, the morphologies of MCs and BCs were strikingly different, with MCs having characteristic descending dendrites and axonal arbours that ramified into L1, whereas BC morphologies were more compact (Buchanan *et al*. 2012). In addition, two types of BCs were found: type 1 with an ascending axon and type 2 with axonal ramifications largely confined to L5. The ascending axons of type‐1 BCs differed from those of MCs in that they did not penetrate L1. Even though the axonal branching pattern for type‐1 cells was unorthodox for BCs, we opted to denote both cell types as ‘BCs’ because they both had short‐term depressing excitatory inputs and a high‐threshold fast‐spiking pattern characteristic of BCs. The existence of these two BC types is in agreement with our previous studies that also identified these two fast‐spiking Pvalb‐positive BC types in L5 of the mouse visual cortex (Buchanan *et al*. 2012; Ferreira *et al*. 2014). Fast‐spiking Pvalb‐positive BCs with ascending translaminar axonal arborizations have also been found in neocortical L6 (Bortone *et al*. 2014), suggesting that cross‐laminar BC inhibition is a general organizational principle of neocortical microcircuits.

### Cell‐specific expression of GluA2

In L5, we found greater GluA2 immunoreactivity in Sst‐positive cells than in Pvalb‐positive cells. Can this be equated directly with MCs and BCs? Many attempts have been made to link molecular expression with the anatomical and electrophysiological features of INs (Markram *et al*. 2004; Ascoli *et al*. 2008; DeFelipe *et al*. 2013; Kepecs & Fishell, 2014). Sst is expressed in all MCs (Wang *et al*. 2004; Toledo‐Rodriguez *et al*. 2005); this is true for assays of protein or mRNA, regardless of neocortical region and layer (Wahle, 1993; Kawaguchi & Kubota, 1996, 1997; Wang *et al*. 2004; Toledo‐Rodriguez *et al*. 2005). Accordingly, Sst is considered as one of the most specific genetic markers (Toledo‐Rodriguez *et al*. 2005). Pvalb is the next most specific of available molecular markers (Toledo‐Rodriguez *et al*. 2005), being primarily associated with fast‐spiking BCs (Cauli *et al*. 1997; Kawaguchi & Kubota, 1997; Dumitriu *et al*. 2007). Although Pvalb and Sst probably predominantly identify BCs and MCs, respectively, it is important to note that no genetic marker known to date unambiguously identifies a single IN type (Markram *et al*. 2004; Ascoli *et al*. 2008; DeFelipe *et al*. 2013; Kepecs & Fishell, 2014).

In an immunolabelling study of monkey visual cortex (Kooijmans *et al*. 2014), Pvalb‐positive INs were reported to label strongly for both GluA2 and GluA3, whereas Sst‐positive INs showed little or no GluA2 labelling (Kooijmans *et al*. 2014). This is the opposite to the findings of the present study obtained in the mouse. However, in the mouse and monkey, different IN classes are defined by different molecular markers (Wahle, 1993; Conde *et al*. 1994; Gonchar *et al*. 2007; Xu & Yao, 2010). This raises the possibility that these species differences are not so much about varying GluA2 expression as they are about differences in IN genetic markers.

### Synapse type‐specific expression of CP‐AMPARs

Pronounced rectification of BC mEPSCs, of uncaging‐induced currents in BCs and of PC‐BC unitary EPSCs supports the suggestion prompted by our immunolabelling that CP‐AMPARs are expressed in BCs but not in MCs or PCs. However, we obtained an incomplete Naspm block of evoked as well as of spontaneous AMPAR‐mediated EPSCs in BCs. One interpretation is that CP and CI‐AMPARs are co‐expressed at excitatory inputs onto BCs, although with CP‐AMPARs dominating. However, several studies have shown incomplete block with Naspm or the related polyamine spider toxin philanthotoxin‐433 for both recombinant CP‐AMPARs (Washburn & Dingledine, 1996; Jackson *et al*. 2011) and for native receptors in cells lacking GluA2 (Koike *et al*. 1997; Sara *et al*. 2011; Studniarczyk *et al*. 2013). Furthermore, synaptic responses in BCs both in paired recordings and with AMPA uncaging were almost abolished at positive voltages by internal spermine. A more parsimonious explanation may be that Naspm cannot fully block CP‐AMPARs under our experimental conditions. Given the known use‐dependence of blockade (Washburn & Dingledine, 1996; Koike *et al*. 1997), additional work would be required to clarify this issue.

Recently, it was suggested that spontaneous and evoked glutamate release activate non‐overlapping populations of receptors (Atasoy *et al*. 2008; Sutton & Schuman, 2009; Sara *et al*. 2011) or can occur preferentially at different sets of synapses (Peled *et al*. 2014). Our data on BCs, however, suggest that glutamate released in an AP‐dependent or independent fashion activates AMPARs with similar properties.

In the present study, we found that CP‐AMPARs were similarly expressed at PC synapses onto both type‐1 and type‐2 BCs. By contrast, we previously reported that PC connections to type‐1 but not to type‐2 BCs differentially expressed presynaptic NMDARs (Buchanan *et al*. 2012). Excitatory inputs to translaminar type‐1 BCs thus have presynaptic NMDARs and postsynaptic CP‐AMPARs, whereas excitatory synapses onto intralaminar type‐2 BCs do not have presynaptic NMDARs, even though they too have postsynaptic CP‐AMPARs. The functional relevance of this interesting dichotomy remains unclear at present.

Other studies, conducted in both the cortex and hippocampus, have also identified the presence of CP‐AMPARs in BCs. Several studies have shown inward rectification and faster kinetics of currents in outside‐out somatic patches from BCs in the rat dentate gyrus, as well as in neocortical fast‐spiking non‐pyramidal cells in the rat frontal cortex (Geiger *et al*. 1995; Koh *et al*. 1995; Angulo *et al*. 1997). Furthermore, inward rectification has been associated with a relatively low abundance of GluA2 mRNA (Geiger *et al*. 1995; Angulo *et al*. 1997). More recently, Wang and Gao (2010) showed that the majority of fast‐spiking INs in the rat prefrontal cortex have inwardly rectifying EPSCs, suggesting the presence of CP‐AMPARs. The existence of CP‐AMPARs in Pvalb‐positive INs in the prefrontal cortex was confirmed by Tao *et al*. (2013), who showed a pronounced inward rectification of evoked EPSCs in adult mice.

It has recently been suggested that CP‐AMPAR expression in INs of the hippocampus reflects the developmental origin of the cells, and may be restricted to those derived from the medial ganglionic eminence (MGE) (Matta *et al*. 2013). Of note, fate‐mapping studies in the cortex have shown that both Pvalb‐positive‐ and Sst‐positive INs originate from the MGE (Wonders & Anderson, 2006; Kessaris *et al*. 2014), albeit primarily from the ventral and dorsal aspects, respectively (Fogarty *et al*. 2007; Wonders *et al*. 2008). Thus, because both L5 BCs and MCs may derive from MGE progenitors, our findings showing that these two IN types have different AMPAR subtypes at their excitatory inputs appear to be at odds with the picture emerging from the hippocampus. However, it is important to note that gene expression profiling has revealed considerable molecular heterogeneity between the dorsal and ventral MGE (Wonders *et al*. 2008). As noted by Matta *et al*. (2013), a purely origin‐dependent rule for the expression of CP‐ *vs*. CI‐AMPARs is probably too simplistic because individual INs have been demonstrated to express CP‐ and CI‐AMPARs at synapses innervated by distinct afferent inputs (Toth & McBain, 1998).

In many cell types, the expression of CP‐AMPARs is developmentally regulated. Unfortunately, no clear patterns emerge from the literature, with different cell types and different brain regions exhibiting different developmental profiles for CP‐AMPARs. Although many studies have suggested a developmental decrease in CP‐AMPAR expression (Kumar *et al*. 2002; Shin & Lu, 2005; Osswald *et al*. 2007; Soto *et al*. 2007), others have shown expression fluctuating with age (Wang & Gao, 2010). Most relevant to our work, CP‐AMPAR expression in Pvalb‐positive L2/3 INs of the mouse visual cortex was recently shown to increase at P31–P34 compared to P17–19 (Lu *et al*. 2014). Our experiments were carried out using tissue from 12‐ to 21‐day‐old mice. Although this age range spans eye opening at P14 (i.e. a key developmental milestone for visual cortex), we found no evidence for developmental changes in BC CP‐AMPAR expression.

### Functional implications in health and disease

What is the functional significance of differential and synapse‐specific CP‐AMPAR expression? In the local circuit, MCs and BCs may act as high‐ and low‐pass filters, respectively (Blackman *et al*. 2013): the strong facilitation of excitatory inputs onto MCs enables delayed‐onset feedback inhibition (Silberberg & Markram, 2007), whereas the short‐term depressing excitatory inputs onto BCs ensure they provide early‐onset feed‐forward inhibition of PCs (Kawaguchi & Kubota, 1997; Buchanan *et al*. 2012). This rapid BC‐mediated feedforward inhibition act perisomatically on principal neurons such as PCs to shorten their integrative time window for excitation (Pouille & Scanziani, 2001; Mittmann *et al*. 2005). In addition, we found that PC‐BC synaptic currents decayed faster that PC‐PC and PC‐MC connections. Our dynamic clamp experiments confirmed the computer model prediction indicating that rapidly decaying CP‐AMPAR synaptic currents result in shorter AP latency and a sharper response duration in BCs compared to the slower CI‐AMPAR currents that were characteristic of excitatory inputs to MCs. These findings suggest that the specific expression of fast CP‐AMPARs at PC‐BC synapses helps temporally sharpen BC‐mediated early inhibition of PCs, further tightening the integration time window in PCs (Pouille & Scanziani, 2001; Mittmann *et al*. 2005). These results are in general agreement with a body of literature showing that excitatory inputs onto BCs tend to have faster kinetics than onto principal neurons (Geiger *et al*. 1999).

Although the difference in kinetics of the two excitatory input types onto BCs and MCs may arise from the differential expression of CP‐AMPARs, other factors, such as subunit composition, auxiliary proteins, glutamate concentration waveform, surface diffusion of astrocytic glutamate transporters, and receptor splice variants (Lomeli *et al*. 1994; Koike *et al*. 2000; Cathala *et al*. 2005; Milstein *et al*. 2007; Kato *et al*. 2010; Jackson *et al*. 2011; Murphy‐Royal *et al*. 2015), are known to determine channel kinetics too, and also probably differ between IN types (Tao *et al*. 2013). In addition, we have previously shown that membrane time constants are faster for BCs than for MCs (Buchanan *et al*. 2012), which also contributes to making BC responses relatively faster. Thus, we do not argue that inputs to BCs necessarily decay faster solely because they have CP‐AMPARs, only that the faster decay is correlated with this specific synapse type. Still, differences in glutamate concentration waveform (Cathala *et al*. 2005) or astrocytic glutamate transporter diffusion (Murphy‐Royal *et al*. 2015) are unlikely explanations for the faster decay kinetics of AMPA uncaging responses in BCs.

Calcium influx can occur via NMDARs, CP‐AMPARs or voltage‐gated calcium channels. Previous work has shown that synapses onto cells that express CP‐AMPARs tend to express few NMDARs and exhibit EPSCs with small NMDAR‐mediated components, whereas those on cells with non‐rectifying EPSCs mediated by CI‐AMPARs tend to exhibit substantial NMDAR‐mediated currents (Angulo *et al*. 1999; Lei & McBain, 2002; Lamsa *et al*. 2007 *a*; Hull *et al*. 2009; Wang & Gao, 2010; Scheuss & Bonhoeffer, 2014). Our findings are in agreement with these results because CP‐AMPAR‐containing PC‐BC synapses appeared to express less NMDAR‐mediated current relative to AMPAR current compared to PC‐PC and PC‐MC connections, which have CI‐AMPARs. However, this difference could potentially also be attributed to space‐clamp error in BCs, if these cells express a different set of dendritic ion channels compared to MCs. We also found that the NMDAR‐mediated current was by no means absent at PC‐BC connections; it was just small relative to the large CP‐AMPAR‐mediated conductance.

Although it is possible that the differential prevalence of NMDARs and CP‐AMPARs simply endows different cell types with alternative routes of calcium entry, it may not be this simple. For example, in supragranular fast‐spiking Pvalb‐positive INs of the mouse, calcium may enter via both routes, with CP‐AMPARs giving rise to a fast calcium influx and causing depolarization that facilitates an additional, slower calcium influx after NMDAR activation (Goldberg *et al*. 2003 *b*). Of note, Goldberg *et al*. (2004) found that synaptically driven calcium elevations in MCs of the visual and somatosensory cortices of mice, which might be expected to rely on NMDARs, were dependent on AMPAR‐mediated depolarization and on activation of T‐type calcium channels, and did not result from activation of NMDARs. Finally, there is also evidence for the existence of a third AMPAR type that is not rectifying yet fluxes calcium (Bowie, 2012).

Differences in CP‐AMPARs, NMDARs and calcium buffering proteins may also underlie cell type‐specific forms of long‐term plasticity (Larsen & Sjöström, 2015). For example, CP‐AMPARs elicit NMDAR independent anti‐Hebbian lonng‐term potentiation at excitatory inputs onto hippocampal INs (Kullmann & Lamsa, 2007; Oren *et al*. 2009; Nissen *et al*. 2010; Szabo *et al*. 2012). The differential expression of CP‐AMPARs among neocortical INs suggests the existence of specific long‐term plasticity rules at PC‐BC and PC‐MC synapses (Larsen & Sjöström, 2015). Such differential plasticity of IN excitatory inputs would have important repercussions for information storage in cortical microcircuits (Lamsa *et al*. 2010). Future work is needed to investigate this possibility.

Dendritic spines serve as biochemical compartments in spiny neurons (Sjöström *et al*. 2008; Maheux *et al*. 2015). BCs do not generally have many dendritic spines. Indeed, spines are found at ∼7‐fold higher density in MCs than in BCs (Kawaguchi *et al*. 2006). BCs, however, express the slow calcium‐binding protein Pvalb (Hof *et al*. 1999), which contributes to their high endogenous calcium‐buffering capacity (Lee *et al*. 2000; Goldberg *et al*. 2003 *a*; Aponte *et al*. 2008). This buffering has been shown to compartmentalize dendritic calcium signals in BCs at the same time as leaving fast CP‐AMPAR‐mediated calcium transients relatively unaffected locally (Goldberg *et al*. 2003 *a*; Aponte *et al*. 2008). BCs might thus additionally need CP‐AMPARs together with the Pvalb calcium buffer to achieve a degree of calcium compartmentalization in the absence of dendritic spines.

In summary, we propose that the synapse‐specific CP‐AMPAR expression may be a general organizational principle of local circuits not just in the neocortex, but also in other brain regions where BCs mediate early‐onset inhibition (Blackman *et al*. 2013). Our findings are important for our understanding of brain functioning not just in health, but also in disease because the disordered regulation of CP‐AMPARs has been associated with a wide range of neurological conditions, such as stroke, epilepsy and neurodegeneration (Cull‐Candy *et al*. 2006; Kwak & Weiss, 2006). In particular, several early studies suggested that CP‐AMPARs contribute to excitotoxicity and cell death (Wright & Vissel, 2012). In this view, known as the GluA2 hypothesis, a pathological switch to the expression of CP‐AMPARs after neurological insult may enhance glutamate toxicity as a result of of elevated calcium influx (Pellegrini‐Giampietro *et al*. 1997). Although much additional work is needed to investigate the potential roles of CP‐AMPARs in long‐term plasticity and in different disease states, the findings of the present study offer a novel perspective on CP‐AMPARs by highlighting just how tightly regulated their synapse‐specific expression is in the neocortex.

## Additional information

### Competing interests

The authors declare that they have no competing interests.

### Author contributions

TL carried out paired recordings, dynamic clamp experiments and AMPA uncaging experiments. JVO carried out immunolabelling and mEPSC recordings. AM, EG, AC, SB, LH and JM carried out the bulk of morphological reconstructions. TL, JVO, MF and PJS analysed the data. PJS carried out the computer modelling, wrote the in‐house data acquisition and analysis software, and designed the dynamic clamp set‐up. PJS and MF conceived the project and designed experiments together with TL and JVO. TL, JVO, MF and PJS wrote the manuscript. All authors have approved the final version of the manuscript and agree to be accountable for all aspects of the work. All persons designated as authors qualify for authorship, and all those who qualify for authorship are listed.

### Funding

This work was funded by MRC CDA G0700188 (PJS), EU FP7 FET‐Open grant 243914 (PJS), CFI LOF 28331 (PJS), CIHR OG 126137 (PJS), CIHR NIA 288936 (PJS), NSERC DG 418546‐2 (PJS), MRC Programme Grant MR/J002976/1 (MF), MRC Project Grant MR/J012998/1 (MF) and Wellcome Trust Programme Grant 086185/Z/08/Z (MF). TL was in receipt of an RI MUHC Studentship and an IPN Returning Student Award. JVO received a UCL PhD Student Impact Award. JM was funded by a Postdoctoral Scholarship from Fonds de recherche du Québec–Santé. The funders had no role in study design, data collection and interpretation, or the decision to submit the work for publication.
